# Non-target-site resistance mechanisms to glyphosate in *Eleusine indica* from Xinjiang cotton fields

**DOI:** 10.3389/fpls.2026.1970303

**Published:** 2026-09-25

**Authors:** Yunlong Li, Yikai Dong, Yue Su, Zongxin Hou, Yibo Wang, Sumei Wan, Wen Chen

**Affiliations:** 1 College of Agriculture, Tarim University, Alaer, China; 2 Key Laboratory of Integrated Pest Management of Xinjiang Corps in Southern Xinjiang, Tarim University, Alaer, China; 3 Key Laboratory of Genetic Improvement and Efficient Production for Specialty Crops in Arid Southern Xinjiang of Xinjiang Corps, Alaer, China

**Keywords:** ABC transporter, cytochrome P450 monooxygenases, *Eleusine indica*, glyphosate, metabolic resistance

## Abstract

Glyphosate resistance in weeds is often mediated by target-site changes in 5-enolpyruvylshikimate-3-phosphate synthase (EPSPS), but non-target-site resistance (NTSR) mechanisms are increasingly recognized as important contributors to field-evolved resistance. Here, we characterized a glyphosate-resistant *Eleusine indica* population collected from cotton fields in Xinjiang, China. Whole-plant dose–response analysis showed that the resistant population GR-R had a 3.36-fold higher GR_50_ than the susceptible population GR-S. GR-R also accumulated substantially less shikimic acid after glyphosate treatment (approximately two-fold lower than GR-S at 24 and 48 hours after treatment), indicating reduced impact on the shikimate pathway. However, sequencing of a conserved *EPSPS* region revealed no substitutions at known resistance-associated residues, and *EPSPS* expression did not differ significantly between GR-R and GR-S. RNA-seq analysis identified 364 differentially expressed genes, with enrichment of oxidative stress, carbohydrate metabolism, amino acid metabolism, hormone signaling, and transporter-related processes. Four upregulated genes associated with herbicide metabolic resistance, *CYP94C1*, *ABCA7*, *ABCG37*, and *ABCG39*, were validated by qPCR. Structural modeling and molecular docking suggested potential interactions between glyphosate and the encoded proteins, with ABCG39 showing the strongest predicted binding among the ABC transporters. These results suggest that glyphosate resistance in GR-R is not driven by common *EPSPS*-based mechanisms but is associated with NTSR involving cytochrome P450 and ABC transporter candidates. Our findings provide a foundation for functional studies of P450- and transporter-mediated glyphosate resistance in *E. indica*.

## 1 Introduction

Weeds are among the most persistent biological constraints to global crop production, competing with crops for light, nutrients, water, and space, and thereby causing substantial yield losses and increasing production costs (Oerke, 2006; Godfray et al., 2010; Soltani et al., 2016). In modern intensive agriculture, chemical weed control remains a major component of weed management because of its high efficacy, broad applicability, and relatively low labor requirement. However, repeated and simplified use of herbicides has imposed strong selection pressure on weed populations, resulting in the rapid evolution of herbicide resistance worldwide (Gaines et al., 2020). To date, at least 548 weed species worldwide have been reported to evolve herbicide resistance (Heap, 2026). The continuing expansion of herbicide resistance threatens the sustainability of current weed management systems and highlights the need to understand the molecular mechanisms by which weeds survive herbicide selection (Yu and Powles, 2014; Riechers et al., 2024; Delye, 2013).

Herbicide resistance mechanisms are generally classified into target-site resistance (TSR) and non-target-site resistance (NTSR) (Délye et al., 2013). TSR usually involves point mutations in the herbicide target protein, target-gene amplification, or target-gene overexpression, all of which reduce herbicide sensitivity at the biochemical site of action (Délye et al., 2013; Yu and Powles, 2014). In contrast, NTSR encompasses a broader and often more complex set of processes, including reduced herbicide uptake, impaired translocation, enhanced metabolic detoxification, vacuolar or apoplastic sequestration (Délye et al., 2013; Gaines et al., 2020; Amrhein and Martinoia, 2021). Compared with TSR, NTSR is frequently polygenic, may confer cross-resistance to herbicides with different modes of action, and is therefore particularly challenging for resistance diagnosis and management (Yu and Powles, 2014; Riechers et al., 2024; Delye, 2013). Among NTSR mechanisms, enhanced herbicide metabolism mediated by cytochrome P450 monooxygenases (P450s), glutathione S-transferases (GSTs), glycosyltransferases (GTs), aldo-keto reductases (AKRs), and ATP-binding cassette (ABC) transporters has received increasing attention (Pan et al., 2021; Luo et al., 2025; Qi et al., 2026; Chen et al., 2025c; Pan et al., 2019; Wang et al., 2025).

Glyphosate is one of the most widely used non-selective herbicides worldwide. Its herbicidal activity results from inhibition of 5-enolpyruvylshikimate-3-phosphate synthase, a key enzyme in the shikimate pathway required for aromatic amino acid biosynthesis (Green, 2018). Inhibition of EPSPS leads to shikimic acid accumulation and disruption of downstream metabolic processes, ultimately causing plant death (Schönbrunn et al., 2001; Gomes et al., 2014). Since its commercialization, glyphosate has been extensively used in orchards, fallow land, non-crop areas, and glyphosate-resistant cropping systems because of its broad-spectrum activity and favorable agronomic characteristics (Green, 2018; Heap and Duke, 2018). However, intensive and repeated glyphosate use has led to the evolution of glyphosate resistance in numerous weed species (Gaines et al., 2020; Heap, 2026). Multiple mechanisms of glyphosate resistance have been reported. TSR mechanisms include amino acid substitutions in *EPSPS*, especially at conserved residues such as Ala100, Thr102 and Pro106, as well as *EPSPS* gene amplification and overexpression (Sammons and Gaines, 2014; Kaundun et al., 2008; Gaines et al., 2010; Chen et al., 2025a; Zhang et al., 2023). NTSR mechanisms have also been implicated in glyphosate resistance. For example, *CYP71AK44* in *Eleusine indica* (He et al., 2024), *ABCC3* in *Lolium* spp (Luo et al., 2025), *AKR4–1* and *ABCC8* in *Echinochloa colona* (Pan et al., 2019, 2021), these NTSR genes have been associated with glyphosate resistance, and heterologous expression of these genes can increase glyphosate tolerance.

Goosegrass (*E. indica*) is regarded as one of the world’s most troublesome weeds (Zhang et al., 2021). Glyphosate has been widely used for its control, however, glyphosate-resistant *E. indica* was first reported in Malaysia and has since been documented in many regions, including China (Lee and Ngim, 2000; Heap, 2026; Li et al., 2022; Chen et al., 2017a). Previous studies have primarily focused on TSR mechanisms (e.g., *EPSPS* point mutations such as Pro106 substitutions and Thr102/Pro106 double mutations, as well as *EPSPS* overexpression and copy number variation) (Kaundun et al., 2008; Zhang et al., 2023, 2021; Li et al., 2022), whereas NTSR has been linked to the overexpression of P450 genes, including *CYP71AK44* (He et al., 2024). Nevertheless, the molecular basis of resistance remains unclear in many field-evolved populations, particularly those lacking *EPSPS* mutations or overexpression (Chen et al., 2025a). Xinjiang is a major cotton-producing region in China where repeated glyphosate use may impose strong selection pressure on *E. indica*, but the resistance status and underlying mechanisms in local populations remain largely unknown. In this study, we characterized glyphosate resistance in field-collected *E. indica* populations from cotton fields in Xinjiang using whole-plant dose–response and shikimic acid accumulation assays, examined *EPSPS* target-site mutations and expression, and explored potential NTSR mechanisms through transcriptome profiling, quantitative PCR (qPCR) validation, homology modeling, and molecular docking. Our results identify upregulated *CYP94C1* and ABC transporter genes as candidate contributors to glyphosate resistance and provide insight into the molecular diversity of NTSR in this important weed species.

## 2 Materials and methods

### 2.1 Plant material and growth conditions

Seeds of three putatively glyphosate-resistant populations of *E. indica* were collected from cotton fields in Xinjiang, China, where glyphosate had been applied historically (**Supplementary Table S1**). A glyphosate‐susceptible (GR‐S) reference population was obtained from a wasteland with no prior herbicide exposure, based on field inspection records. For each population, seeds were randomly harvested from 20 mature plants, air-dried at room temperature (25 ± 2°C) for 7 days, and stored in paper envelopes at ambient temperature until use. All greenhouse experiments were conducted at the Plant Protection Station of Tarim University, under natural photoperiod conditions.

### 2.2 Whole‐plant dose–response assay

To determine the level of glyphosate resistance, seeds were sown in plastic pots (10 seeds per pot) containing a 2:1 (v/v) mixture of organic matter and perlite. At the three‐ to four‐leaf stage, seedlings were sprayed with glyphosate at rates of 0, 135, 270, 540, 1080, or 2160 g acid equivalent (a.e.) ha^−1^ using a HCL 2000F moving‐nozzle spray chamber (Hengchuangli Intelligent Equipment Technology Co., Kunshan, China). Twenty‐one days after treatment (DAT), aboveground biomass was harvested and weighed immediately. The assay was carried out twice, each with three biological replicates per treatment.

### 2.3 Shikimic acid accumulation assay

Since the GR-R population exhibited resistance, further research was conducted on this population. Shikimic acid content was measured as a biochemical indicator of EPSPS inhibition, following a published protocol (Perez-Jones et al., 2007) with minor adjustments. Seedlings at the three‐ to four‐leaf stage were treated with glyphosate at 540 g a.e. ha^−1^. At 0, 24, 48, 72, and 96 hours after treatment (HAT), the second and third leaves were excised, and 0.07 g of fresh tissue was promptly placed into 2‐mL tubes containing 1 mL of 0.25 N HCl. Samples were vortexed, sealed with Parafilm, and subjected to two freeze–thaw cycles (−80°C for 15 min, then 60°C for 15 min), followed by grinding to homogeneity. A 25‐µL aliquot of the homogenate was transferred to a 96‐well plate containing 100 µL of 0.25% (w/v) periodic acid and incubated at 37°C for 60 min. The reaction was terminated by adding 100 µL of 0.6 N NaOH with 0.22 N Na_2_SO_3_, and absorbance was read at 380 nm. Shikimic acid concentrations were calculated from a standard curve. Two independent experiments were performed, each with four biological replicates per time point.

### 2.4 Sequencing of the *EPSPS* gene

To screen for target‐site mutations, genomic DNA was extracted from leaf tissues of nine GR‐R plants that survived the field rate (540 g a.e. ha^−1^) and from untreated GR‐S plants, using the High‐efficiency Plant Genomic DNA Extraction Kit (Tsingke, Xi’an, China). A previously reported primer pair (He et al., 2024) was used to amplify a partial fragment of the *EPSPS* gene (**Supplementary Table S2**). PCR was performed in a reaction mix as described earlier (Chen et al., 2025b), with the following cycling conditions: initial denaturation at 98°C for 10 s; 35 cycles of 98°C for 10 s, 56°C for 5 s, and 72°C for 30 s; and a final extension at 72°C for 7 min. Amplicons were purified with the Trelief^®^ DNA Gel Extraction Kit (Tsingke) and Sanger‐sequenced by Xinjiang Youkang Biotechnology Co. (China). Sequence alignments between GR‐R and GR‐S plants were performed using BioEdit v7.0.9.0.

### 2.5 Quantitative expression analysis of *EPSPS*

Total RNA was extracted from leaf tissues of both populations using the Trelief^®^ Hi‐Pure Plant RNA Kit (Tsingke), and first‐strand cDNA was synthesized with the Goldenstar^®^ RT6 cDNA Synthesis Kit (Tsingke). Quantitative PCR (qPCR) was performed according to previous conditions (Chen et al., 2025b), using the same primer set (**Supplementary Table S2**) as for genomic amplification. Relative transcript levels of *EPSPS* were calculated via the 2^-ΔΔCt^ method, with the eukaryotic translation initiation factor 4A (*eIF‐4*) gene as the internal control (Chen et al., 2017b).

### 2.6 Transcriptome profiling and identification of metabolic resistance candidates

For RNA‐sequencing (RNA‐Seq), total RNA was isolated from aboveground tissues of untreated 3‐ to 4‐leaf seedlings using the RNAprep Pure Plant Kit (Tiangen, Beijing, China), following the manufacturer’s instructions. RNA purity and concentration were assessed with a NanoDrop 2000 (Thermo Fisher Scientific), and integrity was verified using the RNA Nano 6000 Assay Kit on an Agilent 2100 Bioanalyzer. Paired‐end libraries (150 bp) were constructed and sequenced on the Illumina NovaSeq platform (Biomarker Technologies Co., Beijing). Raw reads were processed through the BMKCloud online pipeline (www.biocloud.net) for quality filtering and alignment to the reference genome using Hisat2 (Kim et al., 2015). Functional annotation was performed against Pfam (Finn et al., 2014), KOG/COG (Tatusov et al., 2000; Koonin et al., 2004), Swiss‐Prot (UniProt Consortium T, 2018), KEGG (Kanehisa et al., 2004), and GO (Ashburner et al., 2000) databases. Gene expression levels were quantified as FPKM (Li and Dewey, 2011; Trapnell et al., 2010). Differential expression analysis between GR‐R and GR‐S groups was conducted with DESeq2 (Love et al., 2014), applying thresholds of adjusted *P* < 0.01 and |fold change| ≥ 1.5. Among the differentially expressed genes, those annotated as encoding P450s, GSTs, GTs, AKRs, or ABC transporters were selected as candidates for herbicide metabolic resistance. Candidate genes were further validated by qPCR using the same protocol described in Section 2.5, with primers listed in **Supplementary Table S2**.

### 2.7 Homology modeling and molecular docking

Three‐dimensional structural models of candidate proteins were generated using the SwissModel server (https://swissmodel.expasy.org), and model quality was evaluated with the built‐in assessment tools. Semi‐flexible molecular docking of glyphosate into the predicted active sites was performed using AutoDock Tools v1.5.6. For the receptor, polar hydrogens and Gasteiger charges were assigned; for the ligand (glyphosate), rotatable bonds were defined, non‐polar hydrogens were merged, and atomic charges were added. A docking grid box (126 × 126 × 126 points, spacing 0.375 Å) was generated with AutoGrid4. The Lamarckian genetic algorithm was used with 20 independent runs per conformer. Docking poses were visualized and analyzed in PyMOL v2.3.0.

### 2.8 Statistical analysis

All data were subjected to one‐way analysis of variance (ANOVA) using SPSS v23 (IBM, Armonk, NY, USA). Dose–response curves were fitted with a four‐parameter log‐logistic model in SigmaPlot v10.0 (Chen et al., 2020) to estimate GR_50_ values (the glyphosate rate causing 50% growth reduction). The resistance index (RI) was calculated as the ratio of GR_50_ of the putative resistant population to that of the susceptible population (GR-S).

## 3 Results

### 3.1 Field-collected *E. indica* populations show differential levels of glyphosate resistance

To evaluate the glyphosate sensitivity of field-collected *E. indica* populations, we first compared the whole-plant responses of three putatively resistant populations with those of the susceptible reference population GR-S. Glyphosate treatment caused dose-dependent growth inhibition in all populations, but the magnitude of inhibition differed markedly among biotypes. At 21 DAT, GR-S plants exhibited severe growth inhibition and plant death at 540 g a.e. ha^−1^ and higher rates, whereas the resistant populations retained greater aboveground growth, with the GR-R population showing the strongest survival and biomass retention at the recommended field rate (**Figure 1A****;** **Supplementary Figure S1**).

**Figure 1:**
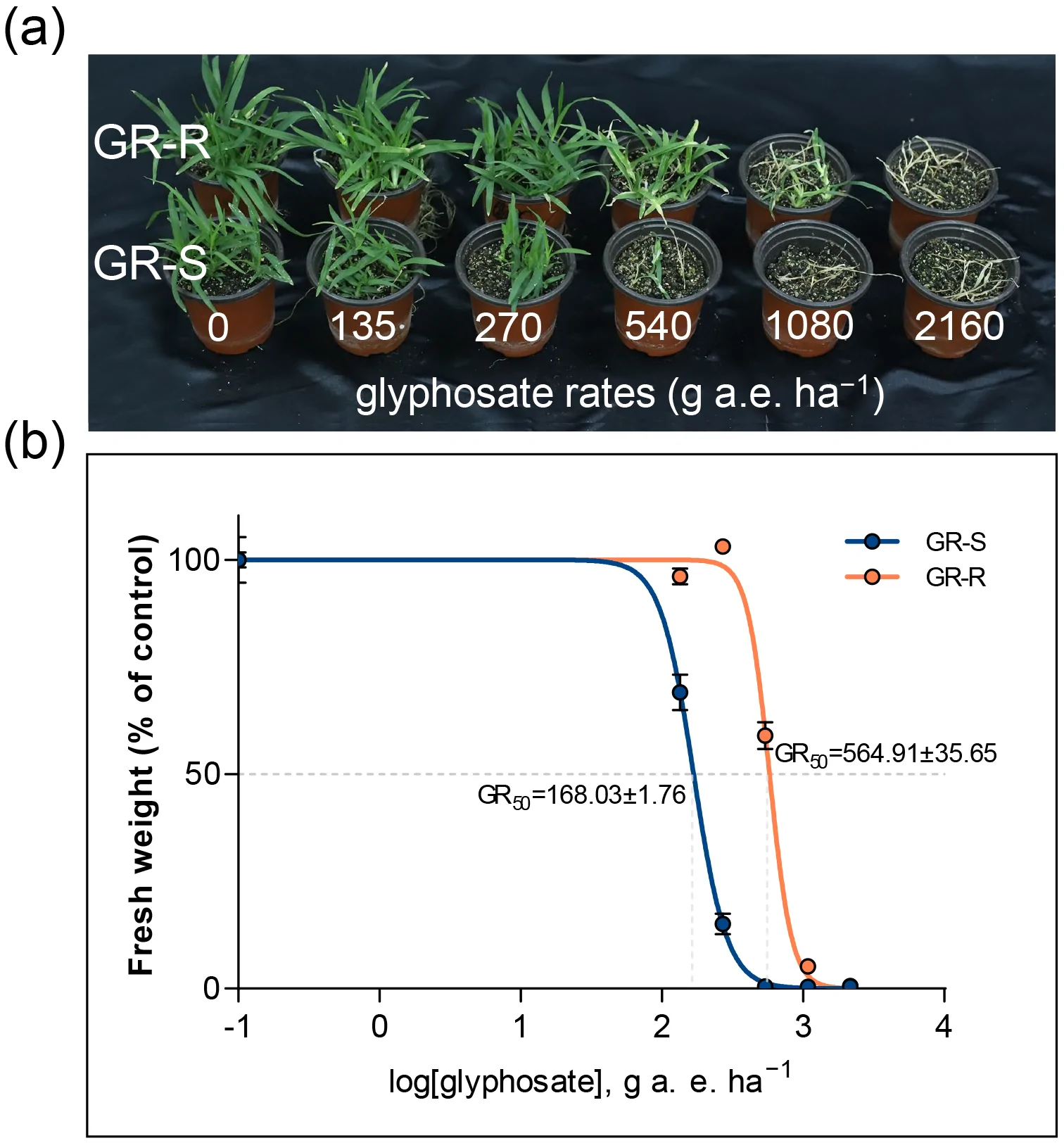
Whole-plant response of GR-S and GR-R *Eleusine indica* populations to glyphosate. **(A)** Representative phenotype of glyphosate-susceptible GR-S and glyphosate-resistant GR-R plants at 21 d after treatment with different glyphosate rates. **(B)** Dose–response curves of GR-S and GR-R plants fitted using a four-parameter log-logistic model.

The dose–response data were well fitted by a four-parameter log-logistic model, with coefficients of determination ranging from 0.9942 to 0.9999, indicating robust model performance across populations (**Supplementary Table S3**). The estimated GR_50_ value for GR-S was 168.03 ± 1.76 g a.e. ha^−1^. In contrast, the GR_50_ values of the three putatively resistant populations were higher, ranging from 257.72 ± 23.38 to 564.91 ± 35.65 g a.e. ha^−1^. Specifically, GR-R_1_ and GR-R_2_ displayed GR_50_ values of 286.00 ± 11.80 and 257.72 ± 23.38 g a.e. ha^−1^, corresponding to RIs of 1.70 and 1.53, respectively. The GR-R population exhibited the highest GR_50_ value, 564.91 ± 35.65 g a.e. ha^−1^, corresponding to a RI of 3.36 relative to GR-S (**Figure 1B****;** **Supplementary Table S3**). Because GR-R showed the highest level of glyphosate resistance among the field-collected populations, this population was selected for subsequent research.

### 3.2 Reduced shikimic acid accumulation confirms reduced glyphosate sensitivity in GR-R

Because glyphosate inhibits EPSPS and thereby causes shikimic acid accumulation, we quantified shikimic acid levels in GR-S and GR-R after treatment with glyphosate at 540 g a.e. ha^−1^. Before glyphosate treatment, shikimic acid contents were similar between GR-S and GR-R, and no significant difference was observed at 0 HAT (**Figure 2**). After treatment, GR-S accumulated large amounts of shikimic acid, reaching high levels at 24 and 48 HAT. In contrast, shikimic acid accumulation in GR-R was substantially lower and remained close to the basal level during the same period (**Figure 2**). Significant differences in shikimic acid content between GR-S and GR-R were detected at 24 and 48 HAT, with GR-S accumulating approximately two-fold higher shikimic acid levels than GR-R. A significant difference was also detected at 72 HAT, although shikimic acid accumulation in GR-S had begun to decline by this time point, possibly due to the typical caused by dying tissue. By 96 HAT, shikimic acid contents were again comparable between the two populations. These biochemical data are consistent with the whole-plant dose–response results and indicate that the GR-R population experiences reduced glyphosate impact after treatment.

**Figure 2:**
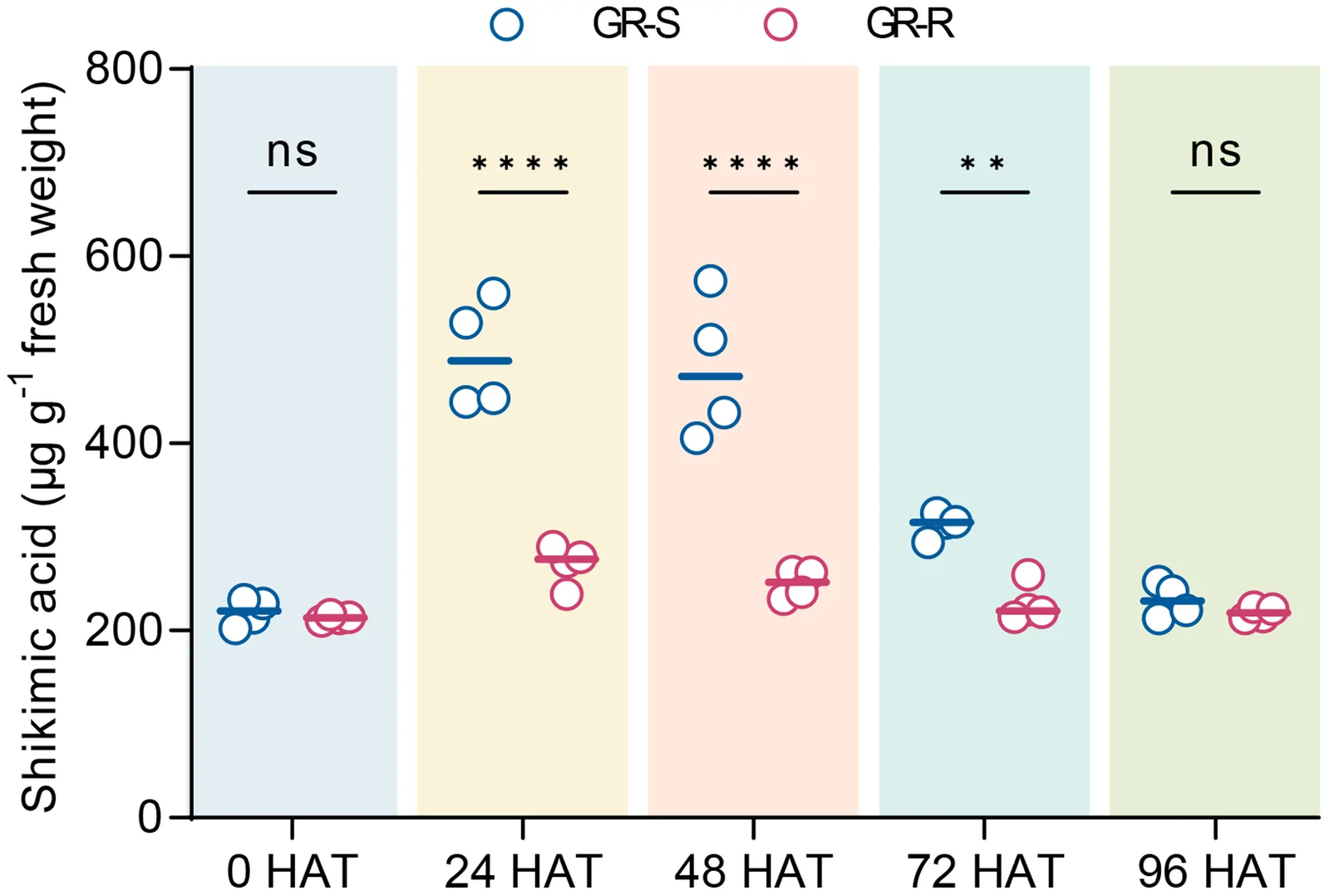
Shikimic acid accumulation in GR-S and GR-R *E. indica* plants after glyphosate treatment. Shikimic acid content in GR-S and GR-R plants was measured at 0, 24, 48, 72, and 96 h after treatment with glyphosate at 540 g a.e. ha^-1^. HAT, hours after treatment. Statistical significance between GR-S and GR-R at each time point was determined using Student’s *t*-test. ns, not significant; **P < 0.01; ****P < 0.0001.

### 3.3 Glyphosate resistance in GR-R is not associated with *EPSPS* target-site mutation or *EPSPS* overexpression

To determine whether resistance in GR-R was associated with target-site mechanisms, we amplified and sequenced a conserved region of the *EPSPS* gene containing amino acid residues commonly associated with glyphosate resistance. Sequence chromatograms from GR-S and GR-R were identical across the amplified EPSPS region, including codons corresponding to Ala100, Gly101, Thr102, Ala103, Met104, Arg105, Pro106, and Leu107 (**Figure 3A**). No amino acid substitutions were detected at Ala100, Thr102 or Pro106, three positions frequently implicated in target-site-based glyphosate resistance. However, we cannot exclude the possibility of mutations outside the sequenced region or *EPSPS* copy number variation. We further examined whether *EPSPS* transcript abundance differed between GR-S and GR-R. qPCR showed that *EPSPS* expression was not significantly different between the two populations (*P* = 0.3299; **Figure 3B**). Together, these results suggest that non-target-site mechanisms may contribute to the resistant phenotype.

**Figure 3:**
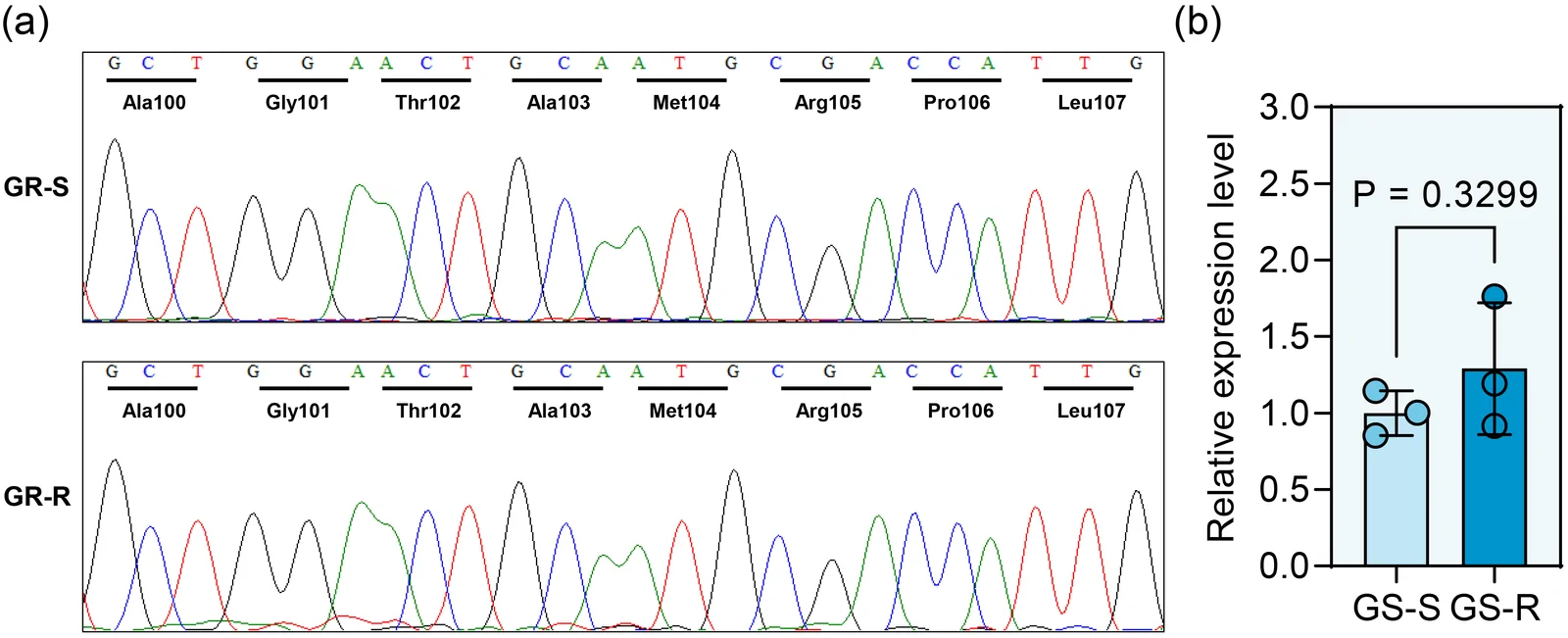
*EPSPS* sequence analysis and expression level in GR-S and GR-R *E. indica* populations. **(A)** Sequence chromatograms of the conserved *EPSPS* region from GR-S and GR-R plants. The amplified region encompasses residues commonly associated with glyphosate resistance (Ala100, Thr102, Pro106). **(B)** Relative *EPSPS* transcript levels in GR-S and GR-R plants determined by qPCR. The *P* value was calculated using Student’s *t*-test.

### 3.4 Transcriptomic profiling identifies candidate genes involved in NTSR

To investigate potential non-target-site mechanisms underlying glyphosate resistance, we performed RNA-Seq analysis using aboveground tissues from GR-S and GR-R seedlings. The sequencing data were of high quality. For each samples, 20.66 to 21.07 million clean reads were obtained per library, corresponding to 6.17 to 6.30 Gb of clean bases. GC content ranged from 51.64% to 52.68%, and Q30 values were consistently high, ranging from 97.64% to 97.81% (**Supplementary Table S4**). Alignment to the reference genome showed that 97.09% to 98.32% of reads were successfully mapped, with 94.90% to 96.19% uniquely mapped and only 2.08% to 2.20% mapped to multiple genomic locations (**Supplementary Table S5**). These results indicate that the transcriptome data were reliable and suitable for downstream differential expression analysis (**Supplementary Figure S2****;** **Supplementary Figure S3**).

We next performed a global functional annotation of the expressed genes. The broad representation of catalytic, binding, and transporter-related functions provided a useful framework for identifying genes potentially involved in herbicide detoxification or transport (**Supplementary Figure S4**). Principal component analysis based on global gene expression profiles showed partial separation between GR-S and GR-R samples along PC1, which explained 38.5% of the total variance, whereas PC2 explained 23.25% of the variance (**Figure 4A**). The PCA showed moderate separation between populations, and we justified the |FC| ≥ 1.5 threshold as a balance between sensitivity and specificity for moderate resistance phenotypes. This separation indicates transcriptomic divergence between the resistant and susceptible populations. Differential expression analysis identified 364 differentially expressed genes (DEGs) between GR-R and GR-S (**Supplementary Figure S5**). Among these, 145 genes were upregulated and 219 genes were downregulated in GR-R relative to GR-S (**Supplementary Figure S5****;** **Figure 4B**). Gene Ontology enrichment analysis of the DEGs showed that enriched biological processes included hydrogen peroxide catabolic process, carbohydrate metabolic process, tryptophan biosynthetic process, response to oxidative stress, abscisic acid-activated signaling pathway, asparagine biosynthetic process, cell wall organization, coenzyme A biosynthetic process, xylan catabolic process, and signal transduction (**Figure 4C**). These enriched categories suggest that oxidative stress responses, primary metabolism, amino acid metabolism, and hormone-related signaling are associated with the GR-R phenotype.

**Figure 4:**
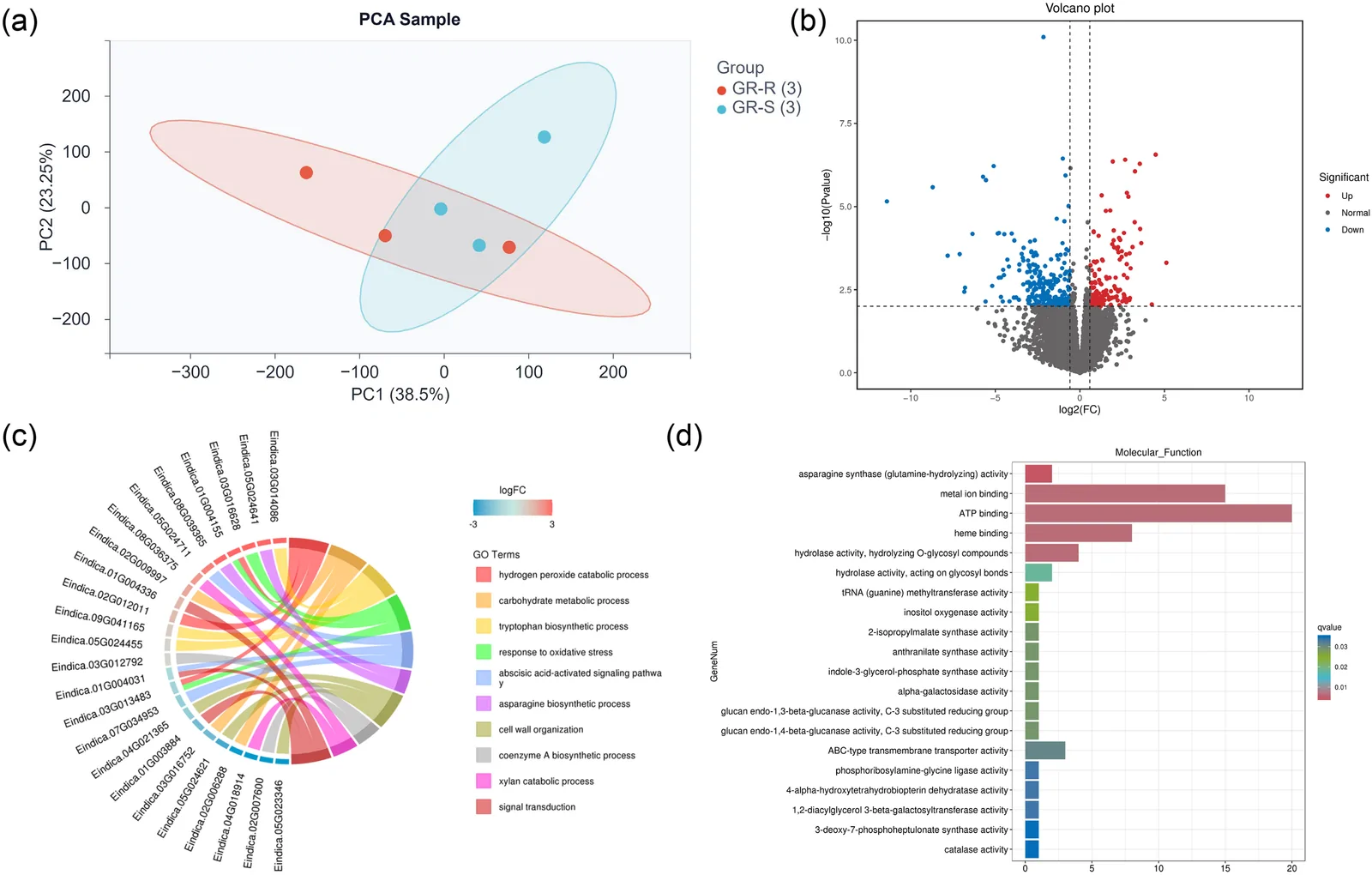
Transcriptome analysis of GR-S and GR-R *E. indica* populations. **(A)** Principal component analysis of global gene expression profiles from GR-S and GR-R samples. **(B)** Volcano plot showing differentially expressed genes between GR-S and GR-R. Red dots indicate genes significantly upregulated in GR-R, blue dots indicate genes significantly downregulated in GR-R, and gray dots indicate genes with no significant difference. **(C)** Chord diagram showing enriched Gene Ontology biological process terms and their associated differentially expressed genes. The outer gene segments indicate log_2_ fold-change values, and colored links connect genes with enriched GO terms. **(D)** Gene Ontology enrichment analysis of differentially expressed genes in the molecular function category. Bar length indicates the number of genes assigned to each GO term, and color indicates the enrichment significance based on the *q* value.

At the molecular function level, enriched categories included ATP binding, metal ion binding, heme binding, hydrolase activity, ABC-type transmembrane transporter activity, oxidoreductase-related activity, and other enzyme activities potentially related to xenobiotic metabolism and transport (**Figure 4D**). Because non-target-site herbicide resistance is frequently mediated by enhanced detoxification, conjugation, sequestration, or transport, we further screened the differentially expressed genes for annotations corresponding to P450s, GSTs, GTs, AKRs, and ABC transporters. This screening identified four upregulated candidate genes potentially associated with glyphosate resistance: *Eindica.04G022553*, annotated as *CYP94C1*, and three ABC transporter genes, *Eindica.06G029920*, *Eindica.04G020888*, and *Eindica.02G011403*, annotated as *ABCA7*, *ABCG37* and *ABCG39*, respectively.

### 3.5 qPCR validation confirms upregulation of *CYP94C1* and ABC transporter genes in GR-R

To validate the RNA-Seq results, we examined the expression of the four selected candidate genes by qPCR using independent biological samples. The qPCR analysis confirmed that all four genes were significantly upregulated in GR-R relative to GR-S, consistent with the RNA-Seq data (**Table 1**). The cytochrome P450 gene *CYP94C1* showed a 1.78-fold increase in RNA-Seq and a 1.85-fold increase by qPCR in GR-R compared with GR-S. Among the ABC transporter genes, *ABCA7* was upregulated 4.71-fold in RNA-Seq and 2.87-fold by qPCR. *ABCG37* was upregulated 3.57-fold in RNA-Seq and 2.88-fold by qPCR, whereas *ABCG39* was upregulated 2.30-fold in RNA-Seq and 4.18-fold by qPCR. All four genes showed significant differences between GR-R and GR-S plants according to the t-test (*P* < 0.05).

**Table 1:** Relative transcription levels of upregulated candidate genes in plants of the glyphosate-resistant GR-R *Eleusine indica* population.

Gene ID	Gene family	Annotation	FC^a^	FC^b^
*Eindica.04G022553*	Cytochrome P450	*CYP94C1*	1.78**	1.85*
*Eindica.06G029920*	ABC transporter	*ABCA7*	4.71**	2.87*
*Eindica.04G020888*	ABC transporter	*ABCG37*	3.57**	2.88*
*Eindica.02G011403*	ABC transporter	*ABCG39*	2.30**	4.18*

FC^a^ indicates the fold change estimated from RNA-Seq data. FC^b^ indicates the fold change determined by qPCR. Fold changes represent transcript levels in GR-R relative to the glyphosate-susceptible GR-S population. Statistical significance between GR-S and GR-R was determined using Student’s *t*-test. **P < 0.01; *P < 0.05.

### 3.6 Structural modeling and molecular docking support potential interactions between glyphosate and candidate proteins

To further assess whether the candidate proteins could potentially interact with glyphosate, we generated three-dimensional structural models for CYP94C1, ABCA7, ABCG37, and ABCG39 and performed semi-flexible molecular docking using glyphosate as the ligand (**Supplementary Figure S6**). Docking simulations showed that glyphosate could be accommodated within predicted binding pockets of all four candidate proteins, with negative binding energies ranging from −3.67 to −4.52 kcal mol^−1^, suggesting thermodynamically favorable interactions (**Figure 5**). For ABCA7, glyphosate docked into a predicted ligand-binding pocket with a binding energy of −3.67 kcal mol^−1^. The docking model indicated that glyphosate formed conventional hydrogen bonds with Lys395 and Glu706 (**Figure 5A**). For ABCG37, glyphosate showed a binding energy of −3.78 kcal mol^−1^. The predicted docking pose placed glyphosate in proximity to several polar residues, including Arg665, Asp206, Asp208, Pro777, and Thr779 (**Figure 5B**). Among these, Arg665 and Thr779 contributed conventional hydrogen-bond interactions, whereas additional carbon hydrogen-bond or polar contacts were predicted with neighboring residues. This interaction pattern suggests that ABCG37 may provide a compatible binding environment for glyphosate. Among the three ABC transporters, ABCG39 showed the strongest predicted interaction with glyphosate, with a binding energy of −4.52 kcal mol^−1^. Glyphosate was predicted to interact with residues including Ser194, Gly195, Lys196, Gln238, and Glu353 (**Figure 5C**). Multiple hydrogen-bond interactions were observed in the predicted binding pocket, consistent with the relatively lower binding energy calculated for ABCG39. Notably, ABCG39 also showed the highest qPCR-validated upregulation among the four candidates, with a 4.18-fold increase in GR-R relative to GR-S (**Table 1**), making it a strong candidate for further functional validation. For the cytochrome P450 protein CYP94C1, glyphosate docked within a predicted catalytic pocket with a binding energy of −4.03 kcal mol^−1^. The docking pose suggested interactions with Lys464 and Asp465, while nearby hydrophobic residues, including Ile462, Cys461, Val460, Leu466, and Leu468, surrounded the ligand-binding region (**Figure 5D**). The presence of polar contacts together with a hydrophobic pocket may facilitate substrate positioning within CYP94C1.

**Figure 5:**
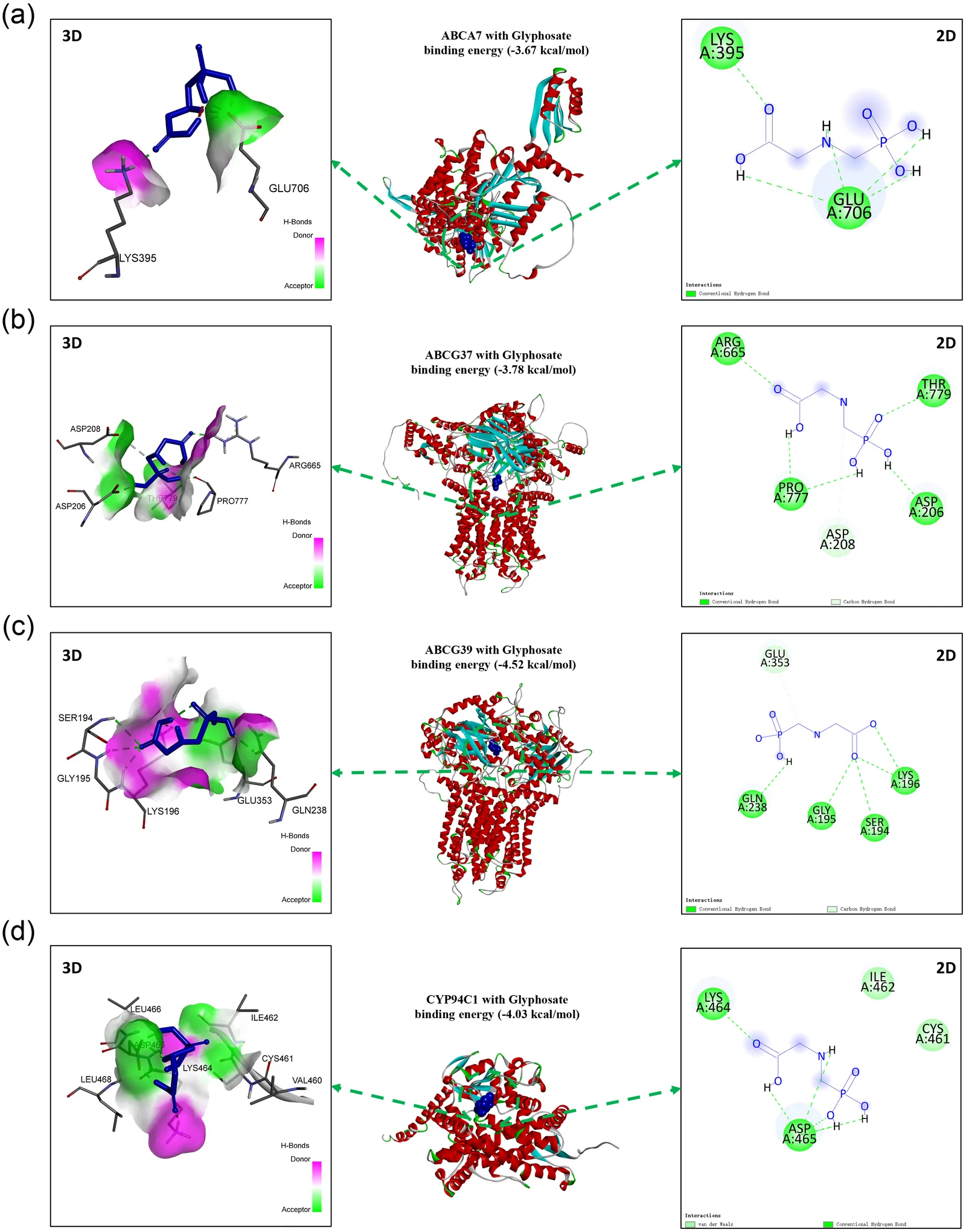
Molecular docking analysis of glyphosate with upregulated candidate proteins in the GR-R *Eleusine indica* population. Docking of glyphosate with **(A)** ABCA7, **(B)** ABCG37, **(C)** ABCG39 or **(D)** CYP94C1. Green dashed lines indicate hydrogen-bond interactions. Donor and acceptor regions in the three-dimensional interaction views are shown in purple and green, respectively. For each protein, the middle panel shows the overall three-dimensional protein structure and the predicted docking position of glyphosate, the left panel shows the local three-dimensional interaction view, and the right panel shows the two-dimensional ligand–residue interaction diagram.

## 4 Discussion

Glyphosate resistance in weeds is often underpinned by diverse and population-specific mechanisms (Powles and Yu, 2010; Gaines et al., 2020; Sammons and Gaines, 2014). In this study, we identified a field-collected *E. indica* population from cotton fields in Xinjiang, China, that displayed reduced sensitivity to glyphosate at both whole-plant and biochemical levels (**Figure 1****;** **Figure 2**). In addition, our results suggest that glyphosate resistance in GR-R is unlikely to be driven by the common *EPSPS*-based mechanisms frequently reported in *E. indica*, and instead are consistent with the possible involvement of NTSR mechanisms (**Figure 3**). The level of resistance observed in GR-R was moderate compared with some highly resistant *E. indica* populations carrying *EPSPS* mutations, *EPSPS* overexpression, or *EPSPS* copy number amplification (Baerson et al., 2002; Kaundun et al., 2008; Gherekhloo et al., 2017; Zhang et al., 2023). Nevertheless, moderate resistance can be agronomically important, particularly when it allows plants to survive recommended field rates and replenish the seedbank (Gaines et al., 2020). In field conditions, even small shifts in glyphosate sensitivity can compromise control efficacy when combined with large population sizes, repeated herbicide exposure, uneven spray coverage, or stress conditions that reduce herbicide performance. The reduced shikimic acid accumulation in GR-R provides biochemical support for the whole-plant phenotype (**Figure 3**). Because shikimate accumulation is a sensitive indicator of EPSPS inhibition by glyphosate (Schönbrunn et al., 2001), the lower accumulation in GR-R suggests that less glyphosate reached or inhibited EPSPS *in vivo*, or that downstream physiological responses to EPSPS inhibition differed between populations. In the absence of EPSPS mutation or overexpression, such a pattern is consistent with reduced glyphosate delivery to the chloroplast, altered intracellular compartmentalization, enhanced efflux, or other NTSR-associated processes.

Transcriptome profiling revealed clear expression differences between GR-R and GR-S and identified biological processes related to oxidative stress, carbohydrate metabolism, amino acid metabolism, hormone signaling, and cell wall organization (**Figure 4**). These functional categories are consistent with the broad physiological perturbations triggered by glyphosate and with the multifactorial nature of NTSR (Delye, 2013; Yu and Powles, 2014; Délye et al., 2013). Glyphosate inhibition of EPSPS disrupts aromatic amino acid biosynthesis and can indirectly affect carbon flow, secondary metabolism, redox homeostasis, and hormone-related pathways (Sammons and Gaines, 2014; Duke and Powles, 2008). Enrichment of hydrogen peroxide catabolism and oxidative stress responses in GR-R suggests that resistant plants may possess an enhanced capacity to buffer glyphosate-associated oxidative damage. Such stress-response remodeling may not itself be sufficient to confer resistance, but it could increase plant survival when combined with mechanisms that reduce active glyphosate concentrations at the target site. However, A limitation of this study is that the transcriptomic comparison was performed between a single resistant population (GR-R) and a single susceptible population (GR-S) collected from different geographical locations. Therefore, some of the 364 differentially expressed genes may reflect population-specific genetic background or local environmental adaptation rather than glyphosate resistance per se.

Among the differentially expressed genes, we identified four upregulated candidates (*CYP94C1*, *ABCA7*, *ABCG37*, and *ABCG39*) with plausible roles in NTSR. P450s are among the most frequently implicated enzyme families in metabolic herbicide resistance, where they catalyze oxidation reactions that initiate detoxification or alter endogenous signaling pathways associated with stress adaptation (Delye, 2013; Yu and Powles, 2014). Several P450s have been functionally associated with resistance to herbicides from different modes of action (Wang et al., 2024; Qi et al., 2026; Han et al., 2021), and recent evidence indicates that P450-mediated processes can also contribute to glyphosate resistance. In *E. indica*, *CYP71AK44* was shown to increase glyphosate tolerance when heterologously expressed, acting together with *EPSPS* overexpression in resistant plants (He et al., 2024). Furthermore, expression of *PfCYP51* in yeast cells significantly enhanced glyphosate tolerance, indicating that *PfCYP51* may be involved in the resistance of *Polypogon fugax* to glyphosate (Chen et al., 2025a). The CYP94 family is known primarily for roles in fatty acid and jasmonate metabolism, especially the oxidative turnover of jasmonoyl-isoleucine and related oxylipin signals (Marquis et al., 2020; Widemann et al., 2015). Thus, *CYP94C1* may contribute to glyphosate resistance through more than one possible mechanism. One possibility is that *CYP94C1* directly modifies glyphosate or glyphosate-associated metabolites (based on the results of molecular docking, **Figure 5D**), although direct oxidative metabolism of glyphosate by plant P450s remains insufficiently established and requires biochemical confirmation. Alternatively, *CYP94C1* may modulate lipid-derived signaling, membrane properties, oxidative stress responses, or wound- and jasmonate-related pathways that influence herbicide tolerance. Because glyphosate exposure can trigger broad metabolic and redox perturbations, altered jasmonate or oxylipin homeostasis may affect the ability of plants to withstand herbicide-induced stress. The observed upregulation of *CYP94C1*, together with the enrichment of oxidative stress-related pathways, is compatible with such a protective or adaptive role (**Figure 4**).

ABC transporters represent another major class of proteins associated with NTSR (Pan et al., 2021). Glyphosate resistance involving altered translocation or sequestration has been reported in multiple weed species, including *Lolium* spp (Luo et al., 2025). and *E. colona* (Pan et al., 2021), where rapid translocation reduces glyphosate movement to sensitive tissues. Here, our finding indicates that *ABCA7*, *ABCG37*, and *ABCG3* were significantly upregulated in GR-R suggests that transporter-mediated processes may contribute to reduced glyphosate sensitivity in this population. Among the three ABC transporter candidates, *ABCG39* is particularly noteworthy because it showed both strong qPCR-validated upregulation and the lowest predicted docking energy with glyphosate among the ABC proteins tested (**Table 1****;** **Figure 5**). Members of the ABCG subfamily are often associated with transport across the plasma membrane and can participate in exporting specialized metabolites, lipids, and xenobiotic-related compounds (Grafe and Schmitt, 2021; Zhou et al., 2024). A recent study showed that the ABCG-type transporter *ArABCG5* confers fomesafen resistance in *Amaranthus retroflexus* (Wang et al., 2026). Therefore, the upregulated ABCG transporters may theoretically mediate the herbicide resistance of weeds. However, the simultaneous upregulation of multiple ABC transporter genes suggests that glyphosate resistance in GR-R may involve a coordinated transport response rather than a single-gene effect.

In conclusion, this study identifies a glyphosate-resistant *E. indica* population from Xinjiang in which resistance is not associated with common *EPSPS* target-site mutations or *EPSPS* overexpression. Transcriptomic, qPCR, and docking analyses highlight *CYP94C1* and three ABC transporter genes as candidate contributors to resistance. These findings broaden our understanding of glyphosate resistance diversity in *E. indica* and provide a foundation for mechanistic studies of P450- and transporter-associated NTSR. Future functional characterization of these candidates will be critical for determining whether they directly mediate glyphosate detoxification or transport, or whether they act as components of a broader stress-adaptive network that enables resistant plants to survive glyphosate selection.

## Data Availability

The RNA-seq data generated in this study, including raw sequencing reads and processed gene-expression data, have been deposited in the NCBI Gene Expression Omnibus (GEO) under accession number GSE347058.
