# Supplementary material for: Non-target-site resistance mechanisms to glyphosate in Eleusine indica from Xinjiang cotton fields
Source: Front Plant Sci. 2026 Sep 25;17:1970303. doi: 10.3389/fpls.2026.1970303 (PMC13649383; doi:10.3389/fpls.2026.1970303)
Supplement: Supplementary file 1 [file SupplementaryFile1.docx]

**Supplementary Information for**

**Non-target-site resistance mechanisms to glyphosate in *Eleusine indica***

**from Xinjiang cotton fields**

Yunlong Li^1, 3†^, Yikai Dong^1, 3†^, Yue Su^1, 3^, Zongxin Hou^1^, Yibo Wang^1^, Sumei Wan^1, 2, 3*^ and Wen Chen^1, 2, 3*^

**Affiliation**

^1^ College of Agriculture, Tarim University, Alaer, 843300, China.

^2^ Key Laboratory of Genetic Improvement and Efficient Production for Specialty

 Crops in Arid Southern Xinjiang of Xinjiang Corps, Alaer, 843300, China.

^3^ Key Laboratory of Integrated Pest Management of Xinjiang Corps in Southern Xinjiang, Tarim University, Alaer, 843300, China.

^†^The first two authors contributed equally to this work.

^*^**Corresponding authors:** Wen Chen (wenchen202410@163.com) and Sumei Wan (sumeiwan8324@163.com).

**Figure S1.** Representative phenotype of glyphosate-susceptible GR-S and glyphosate-resistant (a) GR-R_1_ or (b) GR-R_2_ plants at 21 d after treatment with different glyphosate rates.

**Figure S2.** Simulated distribution of insertion fragment lengths for each sample.

**Figure S3.** Simulation of transcriptome data saturation for each sample.

**Figure S4.** Gene Ontology functional classification of annotated genes in the *E. indica* transcriptome.

**Fig. S5.** Number of differentially expressed genes between GR-S and GR-R.

**Fig. S6.** Quality assessment of the predicted three‑dimensional structures of candidate proteins. Ramachandran plot for the predicted conformation.

**Table S1.** Detailed collecting sites and locations of *Eleusine indica*.

| Collecting locations | | Biotypes | Site | Latitude and longitude |
| --- | --- | --- | --- | --- |
| Xinjiang | Shawan City | GR-S | wasteland | 44.67 °N, 85.28 °E |
|  | Kuitun City | GR-R_1_ | cotton field | 44.49 °N, 84.93 °E |
|  | Shihezi City | GR-R_2_ | cotton field | 44.35 °N, 85.95 °E |
|  | Shihezi City | GR-R | cotton field | 44.54 °N, 85.67 °E |

**Table S2.** Primers used in this study.

| Primer | Direction | Sequence (5′–3′) | Usage |
| --- | --- | --- | --- |
| *EiEPSPS* | Forward | GTGGCAAGTTCCCAGTTGAGA | amplify a partial sequence of the *EPSPS* gene |
|  | Reverse | CAATGGGTCTCTCCCGCATTCTTGG |  |
| *DlEPSPS* | Forward | TCCCAGTTGAGAAGGATGCG | qPCR |
|  | Reverse | CACCAGGTAGCCCTCCGATT |  |
| *DleIF-4* | Forward | CCTACCAAAACGACCACTACGAC |  |
|  | Reverse | ATCACCACCGACCTCCTTGCTC |  |
| *DlCYP94C1* | Forward | GCTTCGACAACTACCCCAAG |  |
|  | Reverse | AGCACTTTGTCGTTGCCTTT |  |
| *DlABCA7* | Forward | GGAAACGCTGACAGGAAGAG |  |
|  | Reverse | TTGGGATCCCCAATTAATGA |  |
| *DlABCG37* | Forward | TGGGACACAGTTGGAACAAA |  |
|  | Reverse | CCGTGTGAATCAGTGAATGG |  |
| *DlABCG39* | Forward | TCCTCCCCTCTGATGTATGC |  |
|  | Reverse | AAGCCAGTCGAGGAACAGAA |  |

**Table S3.** Parameter values of the four-parameter log-logistic equation (Eq. 1)^a^ used to fit the plant growth response (as a percentage of the untreated control) resulting from the different herbicide doses.

| Herbicide | Biotype | Regression parameters | | | | GR_50_ (g a.e. ha^−1^)  (SEM)^b^ | RI^c^ |
| --- | --- | --- | --- | --- | --- | --- | --- |
|  |  | *C* (SEM) | *D* (SEM) | *b* (SEM) | *r*^2^ |  |  |
| glyphosate | GR-S | -0.0024 | 99.9793 | 3.6789 | 0.9999 | 168.03±1.76 | 1.00 |
|  | GR-R_1_ | 1.3949 | 102.5292 | 3.9994 | 0.9978 | 286.00±11.80 | 1.70 |
|  | GR-R_2_ | -2.0004 | 98.208 | 2.6875 | 0.9942 | 257.72±23.38 | 1.53 |
|  | GR-R | 2.4166 | 99.8592 | 7.3437 | 0.9969 | 564.91±35.65 | 3.36 |

^a^ Eq. 1. *y* = *C* + (*D* – *C*)/[1+(*x*/GR_50_)^b^], *C* is the lower limit, *D* is the upper limit, *x* is the herbicide application dose, *b* is the slope of the curve at GR_50_, and *y* is the growth response (as a percentage of the untreated control).

^b^ GR_50_ refers to the herbicide dose resulting in a 50% growth inhibition.

^c^ RI, resistance index. RI was calculated by dividing the obtained GR_50_ value of the other population by that of the GR-S population.

**Table S4.** Statistics of sequencing data. ^1^Sample analysis number; ^2^Total number of pair-end reads in clean data; ^3^Total base count of clean data; ^4^Percentage of total bases in clean data for both G and C bases; ^5^Percentage of bases with clean data mass values greater than or equal to 30.

| Samples^1^ | Clean reads^2^ | Clean bases^3^ | GC Content^4^ | Q30(%)^5^ |
| --- | --- | --- | --- | --- |
| GR-R1 | 21,074,248 | 6,295,038,349 | 51.64% | 97.81% |
| GR-R2 | 20,664,605 | 6,169,090,059 | 52.68% | 97.71% |
| GR-R3 | 21,012,394 | 6,278,769,961 | 52.29% | 97.64% |

**Table S5.** Statistics of sequence comparison results of sample sequencing data with selected reference genomes. ^1^Sample analysis number; ^2^Number of reads compared to the reference genome and percentage of clean reads; ^3^Total base count of clean data; ^4^Number of reads matched to a unique position in the reference genome and percentage of clean reads; ^5^Number of reads compared to multiple locations in the reference genome and percentage of clean reads.

| Sample^1^ | Total Reads^2^ | Mapped Reads^3^ | Uniq Mapped Reads^4^ | Multiple Map Reads^5^ |
| --- | --- | --- | --- | --- |
| GR-R1 | 42,148,496 | 40,923,268 (97.09%) | 39,996,993 (94.90%) | 926,275 (2.20%) |
| GR-R2 | 41,329,210 | 40,635,968 (98.32%) | 39,754,028 (96.19%) | 881,940 (2.13%) |
| GR-R3 | 42,024,788 | 41,269,826 (98.20%) | 40,395,860 (96.12%) | 873,966 (2.08%) |
